# Editorial: Women in inflammatory eye diseases: 2022

**DOI:** 10.3389/fopht.2023.1287568

**Published:** 2023-10-06

**Authors:** María C. Jiménez-Martínez

**Affiliations:** ^1^ Faculty of Medicine, National Autonomous University of México, Mexico City, Mexico; ^2^ Department of Immunology, Institute of Ophthalmology “Conde de Valenciana“, Mexico City, Mexico

**Keywords:** inflammatory eye diseases, central multifocal choroiditis (cMFC), systemic lupus erythematosus (SLE), ocular toxoplasmosis (OT), IL-1b gene polymorphism, biomarkers and treatments, women in science

Welcome to the inaugural Research Topic, “*Women in Inflammatory Eye Diseases, 2022*,” which is dedicated to recognizing the work of women in science, who work daily to comprehend the intricate interplay between ophthalmology and immune-mediated diseases, and emphasizing the research in inflammatory eye diseases associated with women. In this editorial, four interesting articles shed light on the often intricate intersections of systemic and ocular inflammatory manifestations and the search for clinical biomarkers and new treatments.

de Groot et al. conducted a retrospective observational case–control study on patients with central multifocal choroiditis (cMFC), evaluating 104 blood parameters using automated hematocytometry, and correlating the results with the clinical response to systemic corticosteroid-sparing immunomodulatory therapy (IMT). cMFC is an inflammatory eye condition that affects the choroid in the macular region, and, interestingly, this disease impacts a subgroup of young myopic women. The authors demonstrated that there was a significant increase in platelet granularity, suggesting the existence of a potential new biomarker to evaluate corticosteroid-sparing IMT. Despite the need for additional research to evaluate their findings, the utility of this work in low-income countries was enhanced by using a conventional laboratory technique that demonstrates a strong correlation with clinical practice.

Kedia et al. explored the diverse ocular manifestations observed in patients with systemic lupus erythematosus (SLE). Their research highlighted the importance of collaboration between rheumatologists and ophthalmologists in diagnosing SLE when ocular manifestations are the initial autoimmunity symptoms. Integrating ocular symptoms into the early steps of a lupus diagnosis could enable treatment strategies, including targeted immunosuppression or biologic therapy, to improve patient care.

Vergouwen et al. complement the scope of this Research Topic by describing a series of patients with rheumatoid arthritis (RA) and scleritis. The authors examined the changing manifestations of scleritis, particularly in the context of RA treatments. Despite what appears to be a modest increase in the incidence of scleritis over the past decade, the manifestations and complications of scleritis remain unchanged, even with biologic treatments, which has led to a high prevalence of scleral necrosis similar to that in the pre-biologic era. This article shows the significance of the close coordination between medical specialists to monitor and manage scleritis in RA patients effectively.

Araujo et al. analyzed the genetic polymorphisms of the interleukin 1 beta (*IL1β*) gene in a Brazilian population with ocular toxoplasmosis (OT). Their findings showed that the C/C genotype of the *IL1β* gene polymorphism was a protective factor for OT, which is contrary to the findings of other researchers who previously linked this genotype to ocular inflammation. Their research highlights the importance of evaluating immunophenotypes of OT in diverse populations to understand the pathogenesis, clinical outcomes, or even treatment, at a time when precision medicine is becoming more common.

In conclusion, the articles in the Research Topic “*Women in Inflammatory Eye Diseases, 2022*” provided a place for thought-provoking insights into the complex relationships among inflammatory disorders, eye manifestations, and treatment paradigms. The editors hope that this Research Topic will encourage collaboration across medical disciplines, inspire further exploration of inflammatory eye diseases and women-associated ocular disorders research, and contribute to the visibility of the role of women in science.

## Author contributions

MJ-M: Writing – original draft, Writing – review & editing.

